# Picroside II as a Potential Anti-Inflammatory Agent

**DOI:** 10.3390/pharmaceutics18040499

**Published:** 2026-04-17

**Authors:** Yuqian Ren, Zhenchao Liu, Linhai Wei, Yinuo Wang, Yanzhi Wang, Yunliang Guo, Zegang Ma

**Affiliations:** 1 Department of Physiology, School of Basic Medicine, Qingdao University, Qingdao 266071, China; 2 Institute of Brain Science and Disease, Qingdao University, Qingdao 266071, China; 3 School of Pharmacy, Qingdao University, Qingdao 266071, China; 4 College of Materials Science and Engineering, Qingdao University, Qingdao 266071, China; 5 Haide College, Ocean University of China, Qingdao 266071, China; 6 Office of Academic Affairs, Binzhou Medical University, Yantai 264000, China

**Keywords:** inflammation, *Picrorhizae rhizoma*, picroside II, anti-inflammatory

## Abstract

Inflammation, as a basic pathological process, is critically implicated in the pathogenesis and progression of numerous diseases. *Picrorhizae rhizoma* is a type of traditional Chinese medicine with prominent anti-inflammatory effect. And picroside II, a representative iridoid compound, is the major bioactive constituent of *Picrorhizae rhizoma*. Over recent decades, picroside II has garnered extensive research interest owing to its remarkable pharmacological efficacy. Accumulating evidence has validated that picroside II exerts significant anti-inflammatory effects in the prevention and treatment of various systemic diseases. This review comprehensively summarizes and updates the latest research advances of picroside II, systematically elaborating its anti-inflammatory molecular mechanisms, pharmacokinetic profiles, and safety evaluation characteristics. The integrated data and analyses in this review aim to provide solid theoretical support, reliable evidence, and novel insights for the in-depth mechanism research, rational medicinal development, and future clinical translation and application of picroside II.

## 1. Introduction

Inflammation acts as a pivotal pathogenic factor and is widely implicated in the occurrence and development of various chronic and common diseases, including malignant tumors, atherosclerosis, and diabetes [1,2,3,4,5]. Given the critical role of inflammation in disease progression, exploring safe and effective anti-inflammatory agents has become an important direction for disease intervention research. In recent years, natural products have emerged as promising sources of novel anti-inflammatory drugs due to their high biological activities and low toxicity [6,7,8,9].

*Picrorhizae rhizome*, the dried rhizome of *Picrorhiza scrophulariiflora Pennell*, is a traditional Chinese medicine with a long history dating back to the Tang Dynasty [10]. It is mainly distributed in India and regions of Sichuan, Yunnan, Tibet, and the Himalayas in China [10,11]. This herb exhibits significant hepatoprotective, anti-inflammatory, antioxidant and immunoregulatory effects, and is widely used in the treatment of hepatitis, fatty liver, cirrhosis and other liver diseases [6,10,12]. With the in-depth development of modern pharmaceutical research, the chemical composition and pharmacological mechanisms of *Picrorhizae rhizoma* have been gradually clarified.

Modern pharmaceutical research shows that the main chemical components of *Picrorhizae rhizoma* are iridoids, phenylethanol glycosides, and phenolic glycosides [6,10]. Among these components, picroside II is one of the most abundant iridoids isolated from *Picrorhizae rhizoma*, and it is also the most reported bioactive ingredient, whose pharmacological effects have been extensively studied [6,13,14,15]. Previous research has shown that picroside II accumulation has been reported to correlate positively with altitude-related changes in the northwestern Kashmir Himalayas (2740 m a.s.l., 2690 m a.s.l., 1630 m a.s.l.) [16]. Additionally, the same study confirmed that picroside II content was highest in the rhizomes of *Picrorhiza kurrooa*, followed by roots, inflorescences, and leaves [16]. Notably, as a representative iridoid with prominent medicinal value and few reported side effects, picroside II has become a focus of recent anti-inflammatory and hepatoprotective drug development, consistent with the core biological activities of *Picrorhizae rhizom*a [17].

**Figure 1:**
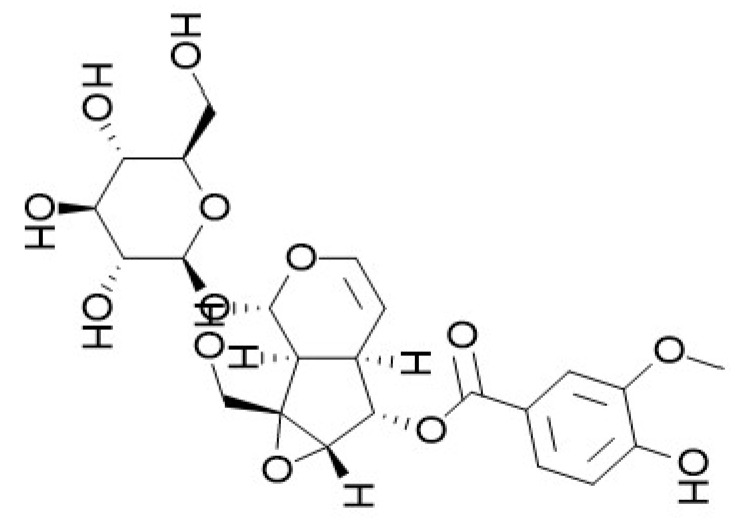
Chemical structure of picroside II. Picroside II (CAS No. 39012-20-9; molecular formula C_23_H_28_O_13_; molecular weight 512.46; synonym: 6-vanilloylcatalpol) is a white amorphous powder with a density of 1.7 ± 0.1 g/cm^3^ and a boiling point of 780.8 ± 60.0 °C. It is readily soluble in water and polar organic solvents including methanol and ethanol, and stable under neutral and weakly acidic conditions. Picroside II is an iridoid glycoside formed via an ester linkage between vanillic acid (4-hydroxy-3-methoxybenzoic acid) and catalpol, an iridoid aglycone [14,20].

Research on picroside II has been garnering increasing attention due to its diverse pharmacological activities. It exerts protective effects on multiple organs, including the brain, heart, and kidney, primarily through various mechanisms such as anti-inflammation, anti-oxidation, and anti-apoptosis [6,17,18,19]. Furthermore, previous studies have shown that picroside II has excellent immunomodulatory effects, which can alleviate inflammatory damage in various diseases by regulating immune cell differentiation, activating inflammatory signaling pathways, and secreting inflammatory factors [6,17,18,19]. Therefore, this review comprehensively summarizes and updates the latest research advances of picroside II, systematically elaborating its anti-inflammatory molecular mechanisms, pharmacokinetic profiles, and safety evaluation characteristics. The integrated data and analyses in this review aim to provide solid theoretical support, reliable evidence, and novel insights for the in-depth mechanism research, rational medicinal development, and future clinical translation and application of picroside II (see Figure 1).

## 2. Pharmacokinetics

Picroside II is a natural product with multiple biological activities. Its bioavailability and drug metabolism in vivo are critical to its therapeutic efficacy and safety. Below is a detailed discussion on the bioavailability and drug metabolic characteristics of picroside II.

### 2.1. Absorption and Distribution

Picroside II exhibits low absolute oral bioavailability. Following intravenous administration, it is rapidly excreted and widely distributed in rats [13,21]. The absorption rate and extent of picroside II after oral administration are highly formulation-dependent [22].

Stability studies confirmed that picroside II remained stable in rat blood over short-term (6 h) and long-term (30 days) periods in different preparations [22]. Significant differences in picroside II content were observed among test materials, including a standardized kutkin mixture, a *P. kurroa* extract, and commercial Picrolax^®^ capsules, which contributed to the marked variations in oral bioavailability.

Key pharmacokinetic parameters of picroside II in rats and dogs after oral or intravenous administration are summarized in Table 1. Notably, picroside II was nearly undetectable in rat urine after oral dosing, suggesting near-complete metabolic clearance [23]. In dogs, intravenous picroside II was rapidly eliminated from plasma, with an average elimination half-life below 30 min [24].

### 2.2. Metabolic Pathways

Gao et al. [20] identified 13 metabolites after oral administration of picroside II in Sprague–Dawley (SD) rats, which were generated mainly through four metabolic pathways: (1) deglycosylation of picroside II to its aglycone, followed by a series of subsequent metabolic reactions; (2) ester bond hydrolysis of picroside II to form vanillic acid, which further underwent sulfation, glycine conjugation, glucosylation, and demethylation; (3) direct glucuronidation of picroside II to produce major metabolites in plasma; and (4) direct sulfation of picroside II. Varun Kumar et al. [25] reported that picroside II is biosynthesized via the degradation of ferulic acid to vanillic acid.

Herbal compounds may alter drug absorption, distribution, and metabolism by inducing or inhibiting metabolic enzymes and transporters, which may reduce therapeutic efficacy and even result in treatment failure [26,27]. Zhou et al. [28] used specific probe substrates to evaluate the effect of picroside II on cytochrome P450 (CYP450) activities in human liver microsomes (0.5–200 μM) and rat liver microsomes (2.5 mg/kg and 10 mg/kg, i.p., for 7 days). Their results indicated that picroside II modulates the activity of multiole CYP enzymes, warranting careful monitoring when picroside II is co-administered with conventional drugs.

In summary, the metabolism of picroside II occurs primarily in the liver and is mediated by the CYP450 enzyme system [28].

### 2.3. Excretion Mechanism

**Figure 2:**
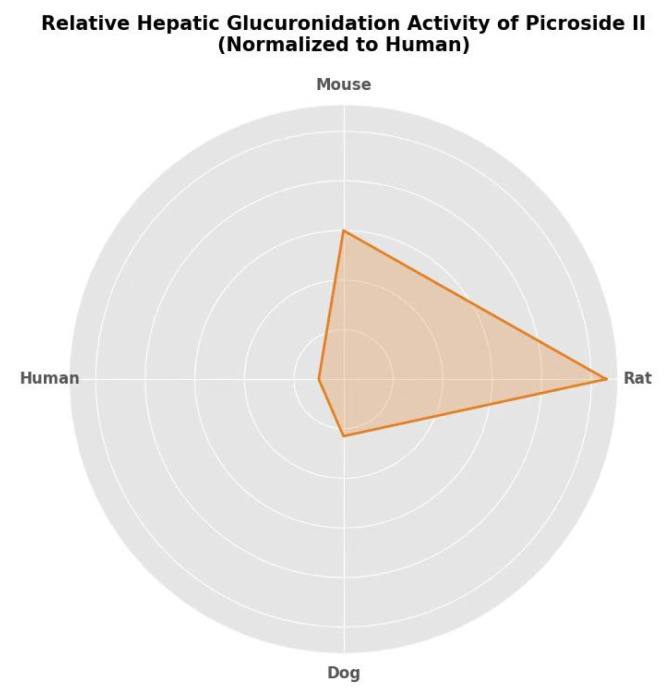
Interspecies comparison of hepatic glucuronidation activity of picroside II.

Studies have shown that picroside II is highly hydrophilic, with poor absorption in the gastrointestinal tract and susceptibility to hydrolysis therein; it is mainly excreted via bile and urine [22]. Consistent with its clinically reported hepatoprotective or neuroprotective effects, picroside II exhibits the highest hepatic uptake and can cross the blood–brain barrier (BBB) following intravenous administration in rats [13]. Li et al. [29] confirmed that the intrinsic clearance rates of hepatic glucosylation of picroside II in rats, mice, and dogs were 10.6-fold, 6.0-fold, and 2.3-fold higher than those in humans, respectively. And the sequence of glucosylation activity of picroside II liver microsomes was rats > mice > humans > dogs [29] (see Figure 2).

## 3. Overview of Anti-Inflammatory Effects of Picroside II

### 3.1. Core Anti-Inflammatory Mechanism of Picroside II

Excessive or dysregulated inflammation is a major driver of tissue damage and the pathogenesis of numerous diseases, including stroke, Parkinson’s disease, atherosclerosis, and cancer [9,30,31,32,33]. Failure to modulate proinflammatory stimuli can lead to persistent chronic inflammation, which exacerbates disease progression [9,34]. Given the critical role of unregulated inflammation in disease development, investigating compounds with anti-inflammatory effects is of great significance for disease intervention—particularly picroside II, a bioactive component derived from *Picrorhizae rhizoma* with proven anti-inflammatory potential, which has become a focus of relevant research. Building on this, the following sections will elaborate on the specific anti-inflammatory mechanisms of picroside II supported by experimental evidence.

#### 3.1.1. NF-κB

The nuclear factor kappa-B (NF-κB) protein was first observed by David Baltimore [35]. There are five distinct subtypes of NF-κB: RelA (p65), RelB, c-Rel, p105, and p100 [36]. Specifically, p65 and p50 play crucial roles in the canonical activation of the NF-κB signaling pathway. NF-κB is found in almost all animal cells and plays a crucial role in cellular inflammatory and immune response [37]. When cells are stimulated or under stress, upstream signals rapidly activate IκB kinase (IKK) to induce IκB phosphorylation, which releases NF-κB from the NF-κB-IκB complex [38]. Mediated by a nuclear localization signal, NF-κB then translocates into the nucleus, binds to specific DNA sequences in gene promoters, promotes the transcription of downstream target genes and protein synthesis, and participates in various pathophysiological processes including infection, inflammation, and immune responses [38,39]. Therefore, inhibition of NF-κB signaling represents one potential mechanism underlying the alleviation of chronic inflammation.

In a recent study, Yao et al. [40] investigated the anti-inflammatory effects of picroside II in a mouse model of dextran sodium sulfate (DSS)-induced ulcerative colitis and in lipopolysaccharide (LPS)-stimulated macrophages. The authors reported that picroside II downregulated the LPS-induced expression of phosphorylated p65 (p-p65) relative to total p65, as well as the levels of key inflammatory mediators including NOD-like receptor protein 3 (NLRP3), caspase-1, and interleukin 1β (IL-1β) in macrophages [40].

Wang et al. [41] demonstrated that picroside II can inhibit NF-κB activation in hyperhomocysteinemia (HHcy)-induced endothelial injury. Wang et al. [42] found that picroside II exerted a protective effect by regulating NF-κB in four different mouse brain injury models. Western blot results showed that Toll-like receptor 4 (TLR4) and NF-κB were significantly downregulated following picroside II treatment. The authors suggested that picroside II may confer neuroprotection against multiple types of brain damage in mice by suppressing excessive inflammatory responses [42]. Huang et al. [43] reported that picroside II inhibited NF-κB and NLRP3 activation in cecal ligation and puncture (CLP)-induced sepsis mice. Western blotting analysis showed that picroside II inhibited CLP-induced NF-κB activation via IκBα, thereby downregulating caspase-1 and IL-1β expression [43].

Similarly, Shen et al. [44] demonstrated that picroside II could inhibit p65 NF-κB in mice with acute lung injury in LPS-stimulated A549 cells. Picroside II may inhibit NF-κB expression in rats with severe acute pancreatitis (SAP), thereby reducing the levels of inflammatory cytokines including IL-1β, IL-6, and tumor necrosis factor-α (TNF-α) [45,46]. Wang et al. [47] confirmed that picroside II also inhibited NF-κB in a rat model of renal ischemia–reperfusion (I/R) injury. Reverse transcription–quantitative polymerase chain reaction (RT-PCR) showed that picroside II attenuated the I/R-induced upregulation of TNF-α, IL-1β, and intercellular adhesion molecule-1 (ICAM-1). Nong et al. [48] similarly verified that picroside II can inhibit NF-κB phosphorylation in chronic constriction injury-induced neuropathic pain. Picroside II blocks the NF-κB pathway by inhibiting NF-κB/p65 phosphorylation and degradation of NF-κB inhibitors (IκB) in astrocytes, while simultaneously inhibiting LPS-induced elevations in IL-1β, IL-6, and TNF-α mRNA and protein levels.

#### 3.1.2. HMGB1-RAGB

The high mobility group box 1 protein (HMGB1) is widely distributed in lymphoid tissues and brain, liver, lung, heart, spleen, kidney, and other tissues. Except for liver and brain tissue, HMGB1 is mainly present in the cytoplasm; in most other tissues, it is primarily located in the nucleus [49,50,51]. Many research studies have shown that extracellular HMGB1 is an effective proinflammatory medium [49,50]. HMGB1 exerts its proinflammatory effects by binding to its specific receptors, including the receptor for advanced glycation end products (RAGE), Toll-like receptor 2 (TLR2), and TLR4. This binding further activates NF-κB, ultimately leading to increased expression and release of multiple inflammatory factors [52]. Li et al. [18] demonstrated that picroside II inhibits HMGB1-RAGE in rats with myocardial ischemia–reperfusion (I/R) injury. Specifically, after picroside II treatment, the expression levels of HMGB1, RAGE, and NF-κB in rats with I/R injury were reduced at the serum, protein, and RNA levels, respectively. Therefore, inhibiting the HMGB1-mediated inflammatory signaling pathway is a promising therapeutic mechanism for picroside II.

#### 3.1.3. MAPK

Mitogen-activated protein kinase (MAPK) serves as a critical signal transducer from the cell surface to the nucleus. MAPK comprises four subfamilies: extracellular regulated protein kinase (ERK), p38 kinases (p38), c-Jun N-terminal kinases (JNK), and ERK5 [53,54]. Multiple stimuli, including growth factors, cytokines, radiation, and osmotic stress, have been shown to activate MAPK signaling pathways [55]. Once activated, MAPK promotes inflammatory responses in mammalian cells, such as enhanced secretion of proinflammatory cytokines [55,56].

NLRP3 is an important mediator that initiates pyroptosis, a form of inflammatory cell death. It can activates caspase-1 and induces the release of abundant proinflammatory cytokines, including IL-18 and IL-1β [57]. Wang et al. [57] found that picroside II (:25 mg/kg and 50 mg/kg in OA mouse model; 20 μM and 50 μM in cellular experiments) inhibited LPS-induced chondrocyte pyroptosis and attenuated osteoarthritis progression in OA mice by regulating the MAPK/NF-κB/NLRP3 signaling pathway.

Accumulating evidence indicates that picroside II can suppress MAPK phosphorylation. Below we summarize the inhibitory effects of picroside II on the MAPK pathway. Lee et al. [58] evaluated the inhibitory effect of picroside II on MAPK signaling in LPS-stimulated human monocytes as a model of chronic obstructive pulmonary disease (COPD). They reported that picroside II inhibited the activation of p38-MAPK, ERK1/2, and IκB pathways [58].

In addition, Yang et al. [59] demonstrated that picroside II significantly reduced the phosphorylation degradation of p38, ERK, JNK, p65, and IκB in RANKL-induced bone loss in vitro and LPS-induced inflammation in vivo. Wu et al. [60] employed UPLC-QTOF-MS, network pharmacology, and experimental validation to investigate the anti-inflammatory mechanisms of the glucoside fraction of *Picrorhiza scrophulariiflora Pennell* extract against colitis. *Picrorhiza scrophulariiflora Pennell* extract significantly inhibited the Akt, p38, ERK, and JNK pathways, both in vivo and in vitro, and picroside II was identified as the major active anti-inflammatory component [60].

#### 3.1.4. JAK-STAT

Janus kinase–signal transducers and activators of transcription (JAK-STATs) are an essential signal transduction mechanism for evaluating anti-inflammatory mechanisms of natural bioactive compounds [61]. The three key components of JAK-STAT signaling include JAKs, STATs, and receptors (which bind chemical signals). JAK1 is a non-redundant core kinase responsible for receptor phosphorylation and dominant in the activation of STAT1/3/5 [62]. Phosphorylation of STATs can drive the transcription of inflammation-related genes, and dysregulated JAK-STAT signaling may lead to a range of diseases, including skin diseases, cancer, and immune disorders [63,64]. Piao et al. [65] demonstrated that picroside II could inhibit the JAK-STAT pathway in a pentobarbital-induced severe acute pancreatitis (SAP) rat model. The results of immunohistochemistry, Western blot, and qPCR showed that after picroside II treatment, the expression levels of phosphorylated JAK2 (p-JAK2) and phosphorylated STAT3 (p-STAT3) in the pancreatic and liver tissues of rats were decreased; additionally, serum levels of inflammatory factors (TNF-α and IL-6) were reduced, while IL-10 levels were increased [65].

#### 3.1.5. Cytokines

Cytokines are small peptides or glycoproteins synthesized and secreted by various tissue cells, primarily immune cells [66]. Based on their biological functions, cytokines can be classified into several families, including interleukin (IL), colony-stimulating factors (CSF), interferon-γ (IFN-γ), TNF, the transforming growth factor-β (TGF-β) family, growth factors (GFs), and chemokines. Generally, cytokines can be divided into pro-inflammatory cytokines and anti-inflammatory cytokines. Pro-inflammatory cytokines are mainly produced by activated macrophages and participate in upregulating inflammatory responses; key examples include IL-1β, IL-6, and TNF-α [3]. Anti-inflammatory cytokines represent a family of immunomodulatory molecules that control the response of pro-inflammatory cytokines [3]. The principal anti-inflammatory cytokines include interleukin-1 receptor antagonists, IL-4, IL-10, IL-11, and IL-13 [66,67].

TNF-α, IL-1β, IL-6, and other inflammatory factors play a key role in inflammatory response, and their excessive release leads to excessive inflammatory amplification and tissue damage [66,67]. Similarly, picroside II reduced the expression of these classic pro-inflammatory cytokines in brain-injured mice [42]. Furthermore, picroside II downregulated mRNA and protein levels of IL-1β, IL-6, and TNF-α in both carbon tetrachloride-induced neuropathic pain mouse models and LPS-stimulated astrocytes [48]. Moreover, picroside II suppressed airway inflammation in a house dust mite (HDM)-induced asthma mouse model. ELISA results revealed that picroside II downregulated Th2-related cytokines (IL-4, IL-5, and IL-13) and upregulated the Th1-related cytokine IFN-γ in bronchoalveolar lavage fluid (BALF) [68].

**Figure 3:**
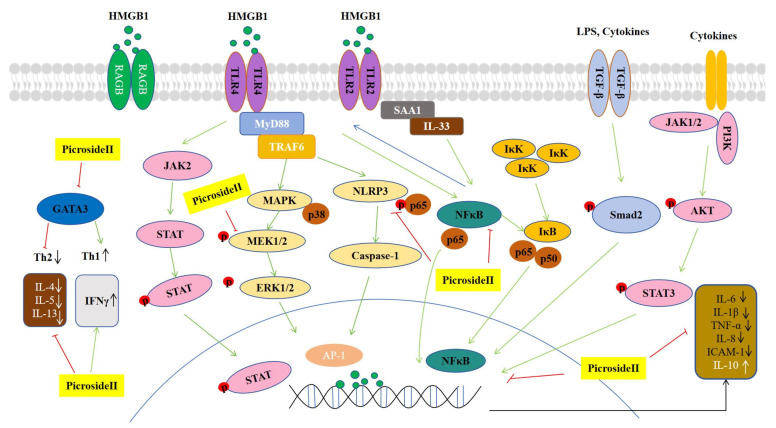
Schematic overview of the anti-inflammatory mechanisms of picroside II. Picroside II exerts anti-inflammatory effects by regulating multiple signaling pathways, including HMGB1/TLR, NF-κB, MAPK, JAK-STAT, and TGF-β pathways. Green arrows indicate activation or promotion, while red arrows indicate inhibition or suppression. Picroside II inhibits the expression of pro-inflammatory cytokines (IL-1β, IL-6, IL-4, IL-5, IL-13, TNF-α, and ICAM-1) and upregulates the anti-inflammatory cytokine IL-10 and IFNγ, thereby suppressing inflammatory responses.

During self-limited inflammatory responses, TGF-β levels in exudates are elevated to exert anti-inflammatory effects [69]. Smads are ubiquitously expressed in various cell types, among which Smad 2 and Smad 4 serve as canonical mediators of the TGF-β signaling response [70]. Picroside II enhanced the phosphorylation of Smad 2 in LPS-induced acute lung injury in RAW264.7 cells [71]. In addition, Western blotting and qPCR results confirmed that picroside II decreased the expression of pro-inflammatory cytokines IL-1β, IL-6, and TNF-α [71] (see Figure 3).

#### 3.1.6. Oxidative Stress

**Figure 4:**
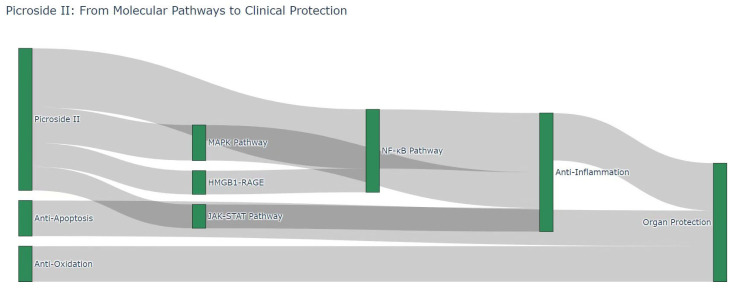
Integrated pharmacological network of picroside II: from molecular pathways to clinical organ protection. The Sankey diagram depicts the mechanistic flow of picroside II’s therapeutic effects. It visualizes the connection between fundamental biological processes (anti-apoptosis, anti-oxidation), intermediary signaling pathways (MAPK, HMGB1-RAGE, NF-κB, JAK-STAT), and the ultimate clinical outcome of multi-organ protection.

Oxidative stress can induce inflammation [72,73]. Under oxidative stress, cells release a series of inflammatory factors, such as TNF-α, IL-1β and IL-6, to trigger inflammation responses [74]. During inflammation, immune cells including neutrophils and macrophages accumulate at inflammatory sites and release excessive reactive oxygen species along with inflammatory mediators, forming a vicious cycle [74,75,76]. Picroside II exerts antioxidant effects by scavenging free radicals in vivo and alleviating oxidative stress-mediated tissue damage [46,77,78,79,80]. Oxidative stress occurs when free radical production exceeds the body’s endogenous antioxidant capacity, thereby causing oxidative damage to tissues and cells. Picroside II attenuates injury by suppressing free radical generation and enhancing the activity of antioxidant enzymes [46,79,80,81] (see Figure 4).

From the above discussion, it can be concluded that picroside II exhibits multiple anti-inflammatory mechanisms. Such pharmacological actions render picroside II a promising anti-inflammatory agent, which holds great value for future research and clinical applications.

### 3.2. Pharmacological Study on Anti-Inflammatory Effects of Picroside II

#### 3.2.1. Cerebral Ischemic

Cerebral stroke is a group of diseases, including ischemic and hemorrhagic stroke, characterized by brain tissue damage resulting from sudden cerebral vascular rupture or occlusion that impairs cerebral blood flow. The mechanisms of cerebral ischemia–reperfusion injury (CIRI) are complex, including oxidative stress, excitatory amino acid toxicity, inflammatory responses, calcium homeostasis imbalance, apoptosis, and more [6]. Natural products with antioxidant and anti-inflammatory properties play an important role in neuronal protection [82].

On the basis of a therapeutic window and dose-finding rationale, the optimal therapeutic dose and time window for cerebral ischemic injury were determined as intraperitoneal injection of 10–20 mg/kg picroside II at 1.5–2.0 h after ischemia onset [83]. Liu et al. [84] demonstrated that intraperitoneal injection of 10 mg/kg picroside II at 1.5–2.0 h after post-CIRI in rats was the optimal dose and timing to achieve therapeutic effect. Pei et al. [85] also found that the optimal dose and time window for picroside II in the treatment of ischemic brain injury was intraperitoneal injection of 20 mg/kg body weight at 1.5 h after ischemia. In a bilateral common carotid artery occlusion (BCCAO)-induced cerebral ischemic model using 30 *Wistar rats*, Zhao et al. identified the optimal regimen of picroside II as 10–20 mg/kg intraperitoneally administered 1.5–2.0 h after ischemia, based on the lowest effective dose and widest therapeutic window [86].

Previous studies have shown that after CIRI in rats, picroside II treatment may inhibit downstream inflammatory factors (e.g., TNF-α and matrix metalloprotein-9 (MMP-9)) via the TLR4-NF-κB signaling pathway, alleviate cerebral edema, and improve brain function [19]. Additionally, picroside II can downregulate the expression of TLR4, NF-κB, and TNF-α; inhibit apoptosis and inflammation in rats with CIRI; and enhance neurobehavioral function [17,87]. Using LPS as an activator of the ERK1/2 pathway and U0126 as an inhibitor, researchers found that picroside II could inhibit the ERK1/2 pathway, reduce the expression of cyclooxygenase 2 (COX2), decrease cerebral infarct volume, and protect neurons in the cortical ischemic area [88,89,90,91].

When 10 mg/kg picroside II was injected into the tail vein of MCAO/R *Wistar rats*, the treatment group showed significantly reduced Bederson’s scores, infarct size, caspase-3 and PARP expression, and apoptotic rates compared with the control group [92]. Picroside II attenuates brain I/R injury in MCAO rats by downregulating the expression of mitochondrial voltage-dependent anion channel 1 (VDAC1), thereby inhibiting the release of endonuclease G (EndoG) from mitochondria to the cytoplasm [93].

Picroside II typically protects rat brain tissue through antioxidant effects. After picroside II treatment, MCAO rats exhibited significantly reduced cerebral infarct volume and brain water content, restored neuronal morphology and structure, and significantly decreased expression of Ras-related *C3* botulinum toxin substrate 1 (Rac1) and NADPH oxidase 2 (Nox2) [94]. Furthermore, we confirmed that picroside II may reduce reactive oxygen species (ROS) levels by downregulating Rac-1 and Nox2 expression, thereby exerting a protective effect on the nervous system. It may also decrease the expressions of Rho-associated coiled-coil protein kinase (ROCK), myosin light chain kinase (MLCK), and MMP2, while enhancing the expression of claudin-5, thus protecting the blood–brain barrier [81]. By establishing an oxygen–glucose deprivation/reperfusion (OGD/R) model using SH-SY5Y cells in vitro, picroside II was shown to improve cell viability, reduce cytotoxicity, and inhibit apoptosis and autophagy in SH-SY5Y cells by suppressing the JNK signaling pathway [95]. Additionally, picroside II can reduce testicular I/R injury in rats by reducing nitric oxide synthesis (NOS) activity, inhibiting apoptosis, and reducing oxidative stress [77].

As a natural product, picroside IIexerts a significant protective effect on cerebral ischemia, particularly through its anti-inflammatory properties s. In summary, numerous studies have shown that picroside II has promising therapeutic potential for CIRI, which is of great significance for the further development of new clinical drugs.

#### 3.2.2. Neurological Diseases

Picroside II has also been extensively studied in other aspects of the nervous system. Wang et al. [42] reported that 20 mg/kg picroside II may restore neural function and provide neuroprotection in brain-injured mice.

In addition, picroside II significantly reversed mechanical allodynia and thermal hyperalgesia induced by chronic constrictive injury and reduced mRNA and protein levels of IL-1β, IL-6, and TNF-α in the spinal cord [48]. The analgesic effect of picroside II was associated with the inhibition of spinal reactive astrocyte-mediated neuroinflammation via the NF-κB pathway in rats with neuropathic pain [48]. Moreover, picroside II (25 μg/mL) acted synergistically with nerve growth factor (NGF) (2 μg/mL) to protect PC12 cells against hydrogen peroxide (H_2_O_2_)-induced oxidative stress [78].

#### 3.2.3. Cardiovascular Diseases

The cardiovascular system, composed of the heart and blood vessels, is a common and critical system affected by diseases that seriously threaten human health [96]. Inflammation plays a central role in the pathogenesis of myocardial I/R injury [97]. Li et al. [18] reported that the protective effect of picroside II on I/R-induced myocardial injury is partly associated with inhibiting the inflammatory response by suppressing the HMGB1-RAGE/TLR2/TLR4-NF-κB signaling pathway. Pretreating cells with wortmannin or LY294002 (specific PI3K inhibitors) abolished the protective effect of picroside II. This study demonstrated that picroside II inhibits I/R-induced cardiomyocyte apoptosis by activating the phosphatidylinositol-3-kinase/protein kinase B/cAMP-response element binding protein (PI3K/Akt/CREB) pathway and regulating the expression of Bcl-2 and Bax [98]. Cardiocytes pretreated with picroside II (50–200 μg/mL) showed increased cell viability in a dose-dependent manner, accompanied by significantly reduced glutathione (GSH) content, decreased superoxide dismutase (SOD) and glutathione peroxidase (GSH-PX) activity, and lower malondialdehyde (MDA) and oxidized glutathione (GSSG) levels [99]. Additionally, picroside II reduced ROS production, improved mitochondrial function, and inhibited hypoxia/reoxygenation-induced cardiomyocyte apoptosis [100]. Picroside II may prevent oxidative damage, inflammation, and apoptosis caused by hyperhomocysteine-induced endothelial injury by regulating the sirtuin1/lectin-like oxidized low-density lipoprotein receptor 1 (SIRT1/LOX1) signaling pathway [41].

#### 3.2.4. Lung Diseases

In acute lung injury models, picroside II significantly reduced TNF-α, IL-1β, and IL-6 levels both in vitro and in mouse tissues, while inhibiting activation of the NF-κB p65 signaling pathway [44]. Picroside II inhibited the expression of LPS-induced neutrophil inflammation and proinflammatory cytokine genes in the lung [71]. Furthermore, picroside II effectively attenuated the expression and secretion of IL-33 induced by serum amyloid A, and this inhibitory effect was mediated by the inhibition of the MAPK, ERK1/2, and NF-κB pathways [58].

#### 3.2.5. Kidney Diseases

Acute kidney injury (AKI) can be induced by renal ischemia–reperfusion injury (IRI). Ren et al. [101] established a bilateral renal IRI-AKI mouse model and evaluated changes in renal microcirculation (Scr, BUN, Cys-C, KIM-1) and inflammatory responses (TNF-α, IL-6) before and after picroside II treatment. Their results demonstrated that picroside II could improve renal microcirculation perfusion impaired by IRI-AKI and attenuate inflammation during AKI [101]. Wang et al. [19] reported that picroside II may protect the ischemic kidney and alleviate renal fibrosis. Inflammation and tissue fibrosis were elevated in rats with renal I/R injury, but were significantly ameliorated after picroside II treatment. Picroside II exerts these effects by inhibiting the TLR4/NF-κB signaling pathway, thereby protecting renal tissue from I/R-induced oxidative stress and inflammation [47]. Furthermore, picroside II treatment attenuated tissue injury and suppressed the expression of cleaved caspase-3 triggered by renal I/R injury [102].

#### 3.2.6. Intestinal Diseases

Picroside II reduces inflammation and enhances immune function in septic mice by inhibiting the activation of the NLRP3 inflammasome and NF-κB pathway [43]. It may also attenuate dextran sodium sulfate (DSS)-induced ulcerative colitis by suppressing the NLRP3 inflammasome and the production of inflammatory factors via the NF-κB signaling pathway [40]. Additionally, picroside II might alleviate intestinal barrier damage caused by pancreatitis. Piao et al. [46] found that picroside II treatment can inhibit the increase in levels of amylase, lipase, MDA, TNF-α, IL-1, IL-6, TLR4, PI3K, Akt, and NF-κB induced by sodium taurocholic acid in rats. Conversely, it increased the levels of SOD, GSPx, catalase (CAT), and IL-10, thereby exerting antioxidant and anti-inflammatory activities.

#### 3.2.7. Liver Diseases

Picroside II exhibits potent hepatoprotective effects due to its high hepatic uptake [13]. In 1983, Kiso et al. [103] identified remarkable anti-hepatotoxic activity of picroside II in primary cultured hepatocytes induced by galactosamine. Picroside II protects hepatocytes from damage and inhibits hepatocyte apoptosis [104]. Picroside II alleviated hepatocyte injury by decreasing the activity of amylase (AMY), alanine aminotransferase (ALT), and aspartate aminotransferase (AST). Meanwhile, it decreased the levels of MDA, TNF-α, IL-1, IL-6, p-JAK2, p-STAT3, Bax, and cleaved caspase 3. Picroside II attenuates severe acute pancreatitis (SAP)-induced hepatocyte injury through its antioxidant and anti-inflammatory effects by modulating the JAK2/STAT3 signaling pathway [65].

Picroside II may represent an effective agent for the prevention and treatment of cholestatic liver disease. Pretreatment with picroside II ameliorated lipid accumulation induced by hepatic steatosis [105]. Furthermore, picroside II may regulate the transporters and enzymes involved in bile acid homeostasis by activating the farnesoid X receptor (FXR), thereby protecting against anti-alpha-naphthylisothiocyanate (ANIT)-induced cholestasis [106]. Jia et al. [107] used RNA sequencing and multi-molecular approaches to investigate the mechanism underlying picroside II-mediated intervention against liver fibrosis in multidrug resistance protein 2 knockout (Mdr2) mice. Their results indicated that picroside II may activate M1 macrophage polarization via the CXCL16-CXCR6 axis to recruit natural killer (NK) cells and prevent the progression of liver fibrosis.

In addition, picroside II exerts protective effects on mouse primary hepatocytes injured by ANIT. It reduces serum biochemical indices, relieves histological damage, and inhibit the phosphorylation of ERK1/2, LKB1, and AMPK in ANIT-induced cholestasis [108]. Picroside II significantly alleviates hepatocyte injury induced by carbon tetrachloride (CCl_4_) and modulates hepatic energy metabolism balance [109]. Xu et al. [80] further demonstrated that prophylactic administration of picroside II protects against D-galactosamine (D-Gal)-induced acute liver injury by reducing oxidative stress, whereas administration after injury may exacerbate CCl_4_-induced chronic liver injury.

#### 3.2.8. Other Diseases

Picroside II exhibits protective effects against severe acute pancreatitis, acute limb I/R injury, cancer, learning, and memory dysfunction. Picroside II reduces the levels of NF-κB, IL-1β, IL-6, TNF-α, and SIRT1, a while increasing SOD and GSH levels in rats with severe acute pancreatitis. When NF-κB was silenced, autophagic activity was suppressed, accompanied by reduced TNF-α and SIRT1 levels. Conversely, NF-κB overexpression enhanced autophagic activity and TNF-α levels, thereby activating SIRT1 [45]. In addition, picroside II at 20 and 40 mg/kg/day significantly attenuated AlCl_3_-induced learning and memory dysfunction in mice [79]. It also inhibits osteoclast differentiation induced by RANKL in vitro and bone loss in vivo [59].

Picroside II markedly decreased GATA3 and Th2 cytokine expression in differentiating Th2 cells, thereby protecting against allergic asthma by suppressing GATA3 expression and Th2 cytokine bias [68]. Picroside II effectively inhibits cancer cell metastasis and angiogenesis both in vitro and in vivo, representing a promising candidate for cancer therapy [110]. A study of 14 iridoid analogues further revealed that picroside II possesses significant anti-hepatitis C virus (HCV) entry and anti-infection activities [111]. Picroside II (5–30 μM) shows promising anti-infectious bursal disease (IBDV) activity and significantly inhibits IBDV replication in a dose-dependent manner for at least 72 h [112].

**Figure 5:**
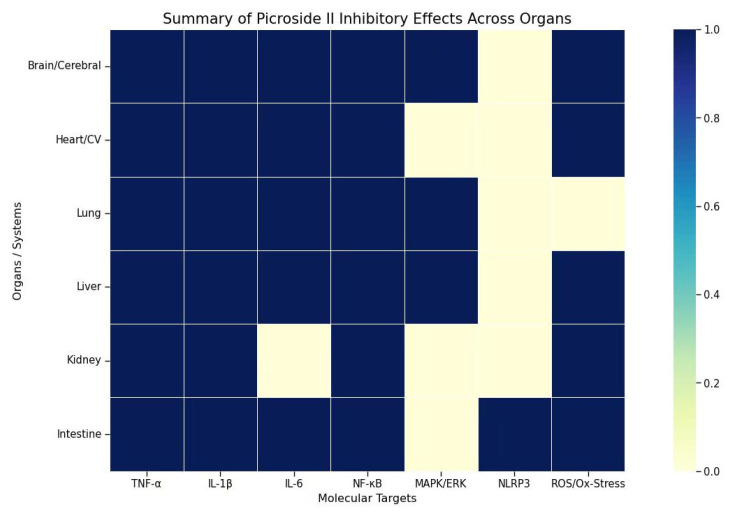
Tissue-specific inhibitory profile of picroside II on key inflammatory and oxidative targets. This heatmap summarizes the inhibitory efficacy of picroside II against various molecular targets (e.g., TNF-α, IL-6, ROS) across different organ systems. The color gradient represents the intensity of the inhibitory effect, with darker blue indicating maximum inhibition (1.0) and light yellow indicating baseline or minimal activity (0.0).

In summary, picroside II acts as a multi-system protective agent, and existing research has mainly focused on its anti-inflammatory, antioxidant, and anti-apoptotic properties. Our review indicates that picroside II exhibits high sensitivity toward inflammatory responses (Table 2) (see Figure 5).

## 4. Safety Evaluation of Picroside II

As a candidate anti-inflammatory bioactive compound with clinical transformation potential, comprehensive and systematic safety evaluation is a core prerequisite for the clinical application of picroside II. This section systematically summarizes the toxicological characteristics, dose-dependent safety effects, long-term safety data, and drug interaction characteristics of picroside II based on existing preclinical studies, to provide a reference for its subsequent clinical development.

### 4.1. Acute/Chronic Toxicity

In acute toxicity studies, picroside II has shown excellent short-term safety in multiple standardized preclinical models. In a 6 h acute toxicity test in mice, oral administration of picroside II did not induce obvious toxic reactions, abnormal behavioral changes, organic damage, or animal death, with no significant abnormalities detected in hematological and serum biochemical indices [22,23]. This suggests that picroside II has a high degree of safety for short-term use.

Chronic toxicity studies are usually conducted to evaluate the safety of a drug over a long period of time after prolonged administration. Recent chronic toxicity studies have shown that picroside II does not cause significant toxic reactions even after prolonged use [22]. A 6-month study in beagle dogs further confirmed no cumulative toxicity of picroside II at 10–30 mg/kg body weight (bw) daily oral doses, with no abnormal physiological or biochemical changes detected [24].

However, the results of these studies may be limited by the dosage, administration period, and species of experimental animals, and therefore more studies are needed to further confirm the safety of long-term use.

### 4.2. Drug Interactions

Drug interactions refer to the interactions that may occur when two or more drugs are used together, which can result in either an enhancement or weakening of the drug’s effects or even adverse reactions. In vitro and in vivo studies have clarified the metabolic pathway and drug interaction risk of picroside II. Its metabolism is mainly mediated by the hepatic CYP450 enzyme system [28]. In vitro incubation experiments with human liver microsomes showed that picroside II (0.5–200 μM) had no significant inhibitory effect on major CYP450 subtypes (CYP1A2, CYP2C9, CYP2C19, CYP2D6, CYP3A4), suggesting a low direct drug interaction risk at conventional effective doses [28].

However, in vivo rat studies confirmed that repeated administration of picroside II can bidirectionally regulate CYP450 activity: 10 mg/kg bw daily doses inhibited CYP2C6/11 activity, while 2.5 mg/kg bw doses induced CYP3A activity. Co-administration with the strong CYP3A inhibitor ketoconazole increased picroside II plasma exposure (AUC) by 2.1-fold, while the CYP3A inducer rifampicin reduced its AUC by 68% [28]. Therefore, caution is required when picroside II is combined with strong CYP3A/CYP2C modulators in clinical practice.

Existing preclinical evidence confirms that picroside II has a favorable overall safety profile. However, further well-designed clinical studies are required to fully validate picroside II’s efficacy and safety in humans.

## 5. Summary and Prospect

Overall, the preclinical evidence summarized in this review consistently demonstrates that picroside II, the core bioactive iridoid of *Picrorhizae rhizoma*, exerts broad-spectrum anti-inflammatory and organ-protective effects across multiple pathological conditions, including cerebral ischemia, cardiovascular disease, neurodegenerative disorders, and hepatic, renal, and pulmonary inflammatory diseases. Unlike conventional anti-inflammatory agents with frequent systemic adverse effects, picroside II exhibits favorable preclinical safety with minimal reported toxic reactions within the effective dose range, highlighting its potential as a promising anti-inflammatory candidate.

However, current research on picroside II has notable critical limitations that restrict its clinical translation. First, most evidence is based on in vitro cell experiments and rodent models, with limited validation in large animals or well-designed clinical trials, leaving translational uncertainty in humans. Second, available pharmacokinetic data are mostly from small animal models, and the absorption, metabolism, optimal therapeutic dose window, and drug interaction characteristics of picroside II in humans remain largely uncharacterized. Third, current mechanistic studies mostly focus on the phenotypic regulation of inflammatory signaling pathways and mediator release, while the direct molecular targets and precise upstream regulatory mechanisms of picroside II have not been fully elucidated. In addition, the heterogeneity of experimental designs (e.g., differences in administration routes, doses, and disease models across studies) reduces the comparability and reproducibility of existing results.

Accordingly, future research should prioritize three core directions: (1) identifying the direct molecular targets of picroside II and clarifying its precise anti-inflammatory mechanisms at the molecular level; (2) conducting standardized pharmacokinetic studies and well-designed clinical trials to validate its therapeutic efficacy, optimal dose, and safety in humans; and (3) systematically evaluating its long-term safety and application risks in special populations (e.g., patients with liver and kidney dysfunction, pregnant women) to support clinical translation.

In summary, this review systematically organizes the research progress on the anti-inflammatory effects and related mechanisms of picroside II, providing a theoretical basis for its subsequent development and application. Despite existing research gaps, picroside II remains a promising anti-inflammatory candidate worthy of further in-depth and standardized investigation.

## Figures and Tables

**Table 1:** Summary of pharmacokinetic characteristics of picroside II.

Species	Administration	Formulation/Dosage	Key Indices	Reference
Male dogs	i.v.	Picroside II (5, 10, 20 mg/kg)	*t*_1_/_2_ < 30 min, detectable plasma concentration at 150 min	[24]
SD rats	Oral	Kutkin (55 mg/kg picroside II)	*C_max_* = 16.66 ± 2.13 ng/mL; *t*_1_/_2_ = 21.72 ± 4.23 h	[21]
Wistar rats	Oral	Iridoid-enriched fraction (50 mg/kg)	*t*_1_/_2_ = 8 h; *C_max_* = 104.62 ± 0.63 ng/mL	[23]
Rats	Oral	*P. kurroa* extract and Picrolax^®^ capsules	*t*_1_/_2_ = 29.50 ± 2.82 h; 33.50 ± 3.08 h; 15.26 ± 1.63 h	[22]

**Table 2:** Summary of the anti-inflammatory effect of picroside II.

Diseases	Model	Dosage	Major Findings	Ref
Allergic asthma	mice and Th2 cells	picroside II (15 and 30 mg/kg, i.p.)	GATA3 and Th2 cytokines	[68]
Acute lung injury	mice and A549 cells	picroside II (mice: 20, 40, 80 mg/kg, i.p.; A549 cells: 40, 80, 160 μg/mL)	IL-1β, IL-6, TNF-α and NF-κB	[44]
Acute lung injury	mice and RAW 264.7 cells	picroside II (0.5/1 mg/kg, i.t.)	TG-Fβ and Smad 2, IL-1β, IL-6, TNF-α	[71]
Brain injury	mice	picroside II (20 mg/kg, i.p.)	TLR4 and NF-κB TNF-α, IL-1β	[42]
Chronic obstructive pulmonary disease	human monocytes	picroside II (20 μM)	MAPK, ERK1/2 and NF-κB	[58]
Cerebral ischemic	rats	picroside II (20 mg/kg, i.v.)	TLR4/NF-κB/TNF-α	[17]
Cerebral ischemic	rats	picroside II (10 mg/kg, i.v.)	TLR4/NF-κB	[87]
Cerebral ischemic	rats	picroside II (20 mg/kg, i.p.)	pMEK1/2, pERK1/2, COX-2	[88]
Hyperhomocysteinemia	HUVECs and mice	picroside II (mice: 10 mg/kg and 60 mg/kg, p.o.; HUVECs: 50 μg/mL, 100 μg/mL, 200 μg/mL)	IL-8, IL-1β, IL-6, TNF-α and NF-κB	[41]
Intestinal barrier injury	rats	picroside II (25 mg/kg, i.v.)	PI3K/AKT/NF-κB, TNF-α, IL-1β, IL-6, IL-10	[46]
Myocardial ischemia–reperfusion	rats	picroside II (10 mg/kg, i.v.)	HMGB1-RAGE/TLR2/TLR4-NF-κB, IL-1β, IL-6, TNF-α, ICAM-1	[18]
Neuropathic pain	rats and primary astrocyte culture	picroside II (10 mg/kg, i.v.)	IL-1β, IL-6, TNF-α and NF-κB	[48]
Osteoarthritis	mice and chondrocytes	Picroside II (25 mg/kg and 50 mg/kg, p.o.); (chondrocytes: 25 μM and 50 μM)	MAPK/NF-κB/NLRP3 caspase-1, IL-18, and IL-1β	[57]
Renal ischemia–reperfusion	rats	picroside II (10 mg/kg, i.v.)	IL-1β, TNF-α, ICAM-1 and TLR4/NF-κB	[47]
Renal ischemia and reperfusion	rats	picroside II (10 mg/kg, i.v.)	TNF-α, IL-1β, IL-10	[19]
Severe acute pancreatitis	rats	picroside II (25 mg/kg, i.p.)	JAK2/STAT3 IL-6, TNF-α, IL-10	[65]
Sepsis	mice and primary macrophages	picroside II (mice: 20 mg/kg, i.v.; macrophages: 200 µg/mL)	NLRP3 and NF-κB pathway IL-1β, IL-6, TNF-α	[43]
Sepsis	mice and BMDM cells	picroside II (mice: 20 mg/kg, i.v.; BMDM: 70 µg/mL)	Caspase 1, NF-κB, GSDMD, NLRP3, IL-6 and TNF-α	[113]
Severe acute pancreatitis	rats	picroside II (25 mg/kg, i.v.)	NF-κB, IL-1β, IL-6, TNF-α, and SIRT1	[45]
Ulcerative colitis	THP-1 cells and mice	picroside II (60 μM)	NLRP3 and NF-κB pathway IL-1β, IL-6, TNF-α	[40]

## Data Availability

No new data were created or analyzed in this study. Data sharing is not applicable.
